# Tumor Necrosis Factor and Interleukin-1β Upregulate NRP2 Expression and Promote SARS-CoV-2 Proliferation

**DOI:** 10.3390/v15071498

**Published:** 2023-07-03

**Authors:** Michinori Ishitoku, Sho Mokuda, Kei Araki, Hirofumi Watanabe, Hiroki Kohno, Tomohiro Sugimoto, Yusuke Yoshida, Takemasa Sakaguchi, Junya Masumoto, Shintaro Hirata, Eiji Sugiyama

**Affiliations:** 1 Department of Clinical Immunology and Rheumatology, Hiroshima University Hospital, Hiroshima 734-8551, Japan; 2 Division of Laboratory Medicine, Hiroshima University Hospital, Hiroshima 734-8551, Japan; 3 Department of Virology, Graduate School of Biomedical and Health Sciences, Hiroshima University, Hiroshima 734-8551, Japan; 4 Department of Pathology, Ehime University Proteo-Science Center and Graduate School of Medicine, Toon 791-0295, Japan

**Keywords:** severe acute respiratory syndrome coronavirus 2 (SARS-CoV-2), coronavirus disease 2019 (COVID-19), Neuropilin-1 (NRP1), Neuropilin-2 (NRP2), tumor necrosis factor alpha (TNFα)

## Abstract

Severe acute respiratory syndrome coronavirus 2 (SARS-CoV-2), which causes coronavirus disease 2019 (COVID-19), utilizes the host receptor angiotensin-converting enzyme 2 (ACE2) and the auxiliary receptor Neuropilin-1 (NRP1) to enter host cells. NRP1 has another isoform, NRP2, whose function in COVID-19 has seldom been reported. In addition, although patients with severe cases of COVID-19 often exhibit increased levels of proinflammatory cytokines, the relationship between these cytokines and SARS-CoV-2 proliferation remains unknown. The aim of this study is to clarify the roles of proinflammatory cytokines in Neuropilin expressions and in SARS-CoV-2 infection. To identify the expression patterns of NRP under inflamed and noninflamed conditions, next-generation sequencing (RNA-seq), immunohistochemistry, quantitative real-time PCR, and Western blotting were performed using primary cultured fibroblast-like synoviocytes, MH7A (immortalized cell line of human rheumatoid fibroblast-like synoviocytes), immortalized MRC5 (human embryonic lung fibroblast), and synovial tissues. To measure viral proliferative capacity, SARS-CoV-2 infection experiments were also performed. NRP2 was upregulated in inflamed tissues. Cytokine-stimulated human fibroblast cell lines, such as MH7A and immortalized MRC5, revealed that NRP2 expression increased with co-stimulation of tumor necrosis factor α (TNFα) and interleukin-1 beta (IL-1β) and was suppressed with anti-TNFα antibody alone. TNFα and IL-1β promoted SARS-CoV-2 proliferation and Spike protein binding. The viral proliferation coincided with the expression of NRP2, which was modulated through plasmid transfections. Our results revealed that proinflammatory cytokines, including TNFα, contribute to NRP2 upregulation and SARS-CoV-2 proliferation in host human cells.

## 1. Introduction

Coronavirus disease 2019 (COVID-19) is an infectious disease caused by severe acute respiratory syndrome coronavirus 2 (SARS-CoV-2). It was first reported in China in 2019 [1], and the number of cases continues to increase worldwide. In about 80% of patients, COVID-19 is a mild disease that resolves spontaneously; other patients can develop severe and fatal conditions [2]. The fatality rate has decreased following the successful development of vaccines and treatments [3,4]. However, new SARS-CoV-2 variants have reduced the efficacy of some vaccines and neutralizing antibodies [5].

SARS-CoV-2 invades the bloodstream through mucous membranes, thereby leading to multiple organ involvement in patients with COVID-19. Its spike (S) protein binds to angiotensin-converting enzyme 2 (ACE2) [6], which is a cell-surface receptor expressed in various cells including human mucosal epithelial cells of the intestinal tract, type II alveolar epithelial cells, vascular endothelial cells, and the oral mucosa [7]. The S protein consists of an S1 subunit containing a receptor-binding domain (RBD), an S2 subunit containing a transmembrane protease serine 2 (TMPRSS2) cleavage site, a fusion peptide (FP) sequence, and a transmembrane sequence. A Furin cleavage site is located between the S1 and S2 sequences. After binding to the host receptor, the S protein is cleaved by TMPRSS2 and Furin, resulting in exposure of the FP site and subsequent entry of the SARS-CoV-2 genetic material into the host cell [8].

Neuropilin (NRP), a receptor for vascular endothelial growth factor, is a transmembrane protein that was originally identified as a neural adhesion molecule [9]. Subsequent studies have shown that it functions as a receptor for Semaphorin, which regulates the development of both the nervous system and inflammatory diseases. NRP has two isoforms, NRP1 and NRP2. NRP1 assists the binding of the S protein to ACE2 [10]; viral replication in vitro is inhibited by anti-NRP1 antibodies, indicating that NRP1 may be an effective target in COVID-19 treatment. NRP2 can bind to the S protein [10], but the role of NRP2 in COVID-19 has seldom been reported.

The majority of studies have reported that proinflammatory cytokines, such as interleukin (IL)-6, tumor necrosis factor α (TNFα), and IL-1β, are related to COVID-19 pathogenesis [11,12]. IL-6 is associated with the severity of COVID-19 [13], and anti-IL-6 receptor antibodies, such as tocilizumab and sarilumab, can alleviate the ordinal scale of symptoms [14,15]. TNFα expression is elevated in many autoimmune lesions, and TNF inhibitors have been approved for the treatment of several autoimmune and inflammatory diseases [16,17,18,19,20]. In 2020, the Global Rheumatology Alliance reported that patients with rheumatic diseases, such as rheumatoid arthritis (RA), who were being treated with TNFα inhibitors had a reduced risk of hospitalization from COVID-19 [21]. Moreover, IL-1β is also associated with COVID-19 pathogenesis, and the NOD-like receptor family pyrin domain-containing 3 (NLRP3) inflammasome, which promotes the production of active IL-1β, is activated in COVID-19 patients [22]. To the best of our knowledge, though these proinflammatory cytokines are related to COVID-19 pathogenesis, the relationship between these molecules and SARS-CoV-2 proliferation has not been reported.

We previously reported that ACE2 expression occurs in synovial tissues and is upregulated by IL-6 [23], which implies that synovial tissues and cells are one of the suitable specimens for the analysis of host receptor regulation under inflammatory conditions during SARS-CoV-2 infection. Here, we aim to elucidate the effect of proinflammatory cytokines on NRP2 expression and to clarify the influence of these cytokines on SARS-CoV-2 proliferation. Our data reveal that proinflammatory cytokines contribute to NRP2 upregulation and SARS-CoV-2 proliferation in host human cells.

## 2. Materials and Methods

### 2.1. Ethics

This study was approved by the Clinical Ethics Committee of Hiroshima University Hospital, Hamawaki Orthopaedic Hospital, and Dohgo Spa Hospital (approval no. E-668; 1 February 2017). Experiments were performed in accordance with approved guidelines. Synovial tissues were collected from patients with RA or osteoarthritis (OA) who had undergone synovectomy or total joint replacement, after obtaining informed written consent.

### 2.2. Next-Generation RNA Sequencing (RNA-seq)

RNA library preparation, sequencing, mapping, and gene-expression analysis were performed using DNAFORM (Yokohama, Kanagawa, Japan), as previously reported [24]. Gene expression was normalized to that of housekeeping genes. Relative expression levels were calculated using the following formula: log_2_[(gene read count per sample)/(total average count of the gene)]. The difference in the mean relative expression level between IL-1β-stimulated and control specimens was then calculated.

### 2.3. Preparation of Primary Cultured Fibroblast-like Synoviocytes (FLS), the MH7A Cell Line and the Lung MRC5 Cell Line

For the culturing of FLS, synovial tissue from RA patients was minced with 1 mg/mL ROCHE collagenase/dispase (Sigma-Aldrich, Tokyo, Japan) in phosphate-buffered saline (PBS; pH 7.2) for 1 h at 37 °C, filtered, washed, diluted, and cultured. After culturing, the supernatant was replaced to remove non-adherent cells. Adherent FLS were split at a ratio of 1:3 with 80% confluent and then passaged. FLS were used for the experiments at passages 3–5 and seeded at 1 × 10^5^/mL. They were cultured in Dulbecco’s modified Eagle’s medium (DMEM) (FUJIFILM Wako Pure Chemical Co., Osaka, Japan) containing 10% fetal bovine serum (FBS) (Sigma-Aldrich) and penicillin/streptomycin (FUJIFILM Wako Pure Chemical Co.). Immortalized rheumatoid fibroblast-like synoviocytes (MH7A cells) were obtained from KISSEI Pharmaceutical Co., Ltd. (Matsumoto, Japan). MH7A cells were cultured in Roswell Park Memorial Institute 1640 (RPMI 1640; FUJIFILM Wako Pure Chemical Co.) medium containing 10% FBS and penicillin/streptomycin. Immortalized MRC5 cells, which are human embryonic lung fibroblasts, were provided by the JCRB Cell Bank (No. JCRB1615, Tokyo, Japan) and cultured in DMEM containing 10% FBS and penicillin/streptomycin. The cells were seeded at 1 × 10^5^/mL and incubated at 37 °C under 5% CO_2_. Prior to analysis, the cells were placed in DMEM containing 0.5% FBS for serum starvation for >6 h before the addition of recombinant human cytokines. Then, FLS, MH7A cells and immortalized MRC5 cells were stimulated with recombinant human IL-6 (100 ng/mL), recombinant human soluble IL-6 receptor alpha (sIL-6Rα) (100 ng/mL), recombinant human TNFα (50 ng/mL), recombinant human IL-17 (50 ng/mL), and recombinant human IL-1β (10 ng/mL) (BioLegend, San Diego, CA, USA) or inhibited with anti-human TNFα antibody (5 µg/mL, mouse monoclonal, clone: 28401) and anti-human IL-1β antibody (1 µg/mL, mouse monoclonal, clone: 2805) (R&D Systems, Minneapolis, MN, USA). Mouse IgG1 isotype control (clone: 11711) was also purchased from R&D Systems.

### 2.4. RNA Isolation and Quantitative Real-Time PCR (RT–qPCR)

Total RNA was extracted and purified from cultured cells using TRIzol reagent (Life Technologies, Carlsbad, CA, USA) followed by cDNA synthesis using the PrimeScript RT Reagent Kit with gDNA Eraser (Takara Bio, Kusatsu, Japan). RT–qPCR using Brilliant II SYBR Green QPCR Master Mix (Agilent, Santa Clara, CA, USA) was performed using a CFX Connect Real-Time PCR Detection System (Bio-Rad Laboratories, Hercules, CA, USA). The upstream and downstream primer sequences for ACE2 were 5′-GATTCTTTTTGGGGAGGAGGA-3′ and 5′-CTCCGGGACATCCTGATG-3′, respectively. For NRP1, the upstream and downstream primer sequences were 5′-ACAACGGCTCGGACTGGAAGA-3′ and 5′-GTAGATCCTGATGAATCGCGTG-3′, respectively. For NRP2, the upstream and downstream primer sequences were 5′-TCTCCTACAGCCTAAACGGCA-3′ and 5′-GGTCAAACCTTCGGATGTCAG-3′, respectively. The upstream and downstream primer sequences for human glyceraldehyde 3-phosphate dehydrogenase (GAPDH), which was used as an internal control, were 5′-AAGGTCATCCCAGAGCTGAA-3′ and 5′-CTGCTTCACCACCTTCTTGA-3′, respectively.

### 2.5. Immunohistochemistry (IHC) Staining for Formalin-Fixed Paraffin-Embedded (FFPE) Synovial Tissues

Fluorescent IHC staining was conducted for FFPE synovial tissues collected from RA and OA specimens, as previously reported [24]. The following primary antibodies were used: anti-NRP1 antibody (rabbit monoclonal, clone EPR3113; Abcam, Cambridge, UK), anti-NRP2 antibody (rabbit monoclonal, clone D39A5; Cell Signaling Technology, Tokyo, Japan), anti-human cadherin-11 (CDH11) antibody (goat polyclonal; R&D Systems Inc.), anti-ACE2 antibody (rabbit polyclonal; Bioss Antibodies Inc., Woburn, MA, USA), and anti-TMPRSS2 antibody (rabbit polyclonal; Proteintech, Tokyo, Japan). The following secondary antibodies were used: Alexa Fluor 405-conjugated anti-goat immunoglobulin G (IgG) (host: donkey; Abcam) and Alexa Fluor 647-conjugated anti-rabbit IgG (host: donkey; Abcam). The slides were quenched using the Vector TrueVIEW Autofluorescence Quenching Kit (Vector Laboratories, Burlingame, CA, USA). Nuclear staining was performed with acridine orange (Abcam). Subsequently, glass slides were treated with VECTASHIELD Vibrance Antifade Mounting Medium (Vector Laboratories). Fluorescent images were obtained using a digital microscope VHX-7000 (KEYENCE, Osaka, Japan).

### 2.6. Luciferase Assay

After transfecting with the luciferase reporter plasmids, the cells were incubated for 24 h. The cells were transfected with specificity protein 1 (SP1) and NF-κB (p65) and then lysed and measured using the Dual-Glo Luciferase Assay System (Promega, Madison, WI, USA) in a 96-well plate. Luminescence intensities were determined using a SpectraMax iD3 system (Molecular Devices, San Jose, CA, USA).

### 2.7. Western Blotting

Recombinant proteins and cell lysate proteins were detected using Western blotting. Before harvesting, MH7A cells and FLS were washed with PBS. The cells were homogenized in 2% sodium dodecyl sulfate sample buffer using BioMasher II (Nippi Inc., Tokyo, Japan). The samples were then centrifuged for 5 min at 15,000× *g* and 20–25 °C. Proteins were processed using a SuperSep Ace 10% precast gel (FUJIFILM Wako Pure Chemical Co.) and transferred onto a polyvinylidene fluoride membrane. The membranes were probed with anti-β-actin (mouse monoclonal, clone AC-15; Sigma-Aldrich), anti-NRP1 (rabbit monoclonal, clone EPR3113; Abcam), and anti-NRP2 (rabbit polyclonal; Sigma-Aldrich) antibodies. Rabbit sera against anti-nucleocapsid (N) protein were prepared by immunizing rabbits with the N-terminal domain of SARS-CoV-2 N protein expressed and purified in E. coli. Horseradish peroxidase-conjugated secondary antibodies (Cell Signaling Technology) were then added. Horseradish peroxidase activity was detected using ECL prime reagents (Cytiva, Tokyo, Japan), followed by imaging using an Image Quant LAS 500 (Cytiva) system.

### 2.8. Plasmid Construction and Transfection into MH7A Cell Line

To prepare the recombinant spike (S) protein, a pEU-GST-S1(318–685) plasmid was constructed using custom gene products (Genscript, Piscataway, NJ, USA). The custom-synthesized gene was inserted into a pEU-E01-glutathione-S-transferase (GST) vector (Cell Free Science, Matsuyama, Japan) containing an SP6 promoter. The sequence of the SARS-CoV-2 S protein (Wuhan-Hu-1) corresponded to GenBank accession no. MN908947.3. For the overexpression experiments, the pCMV3 control vector, pCMV3-human SP1, and pCMV3-human NRP2 plasmids were purchased from Sino Biological Inc. (Wayne, PA, USA). The pcDNA3-human p65 construct was prepared. Briefly, full-length cDNA of RelA (p65) (*RELA*; NM_021975.4) was harvested using PCR and inserted into the pcDNA3 vector (Invitrogen, Carlsbad, CA, USA), as previously described [25]. For knockdown experiments, the pLKO.1-puro-shRNA control vector and pLKO.1-puro-shRNA against NRP2 (TRCN0000063311) were purchased from Sigma-Aldrich. For the luciferase assay, a reporter plasmid was constructed using custom gene products (GenScript, Piscataway, NJ, USA). The pGL4.16 vector was purchased from Promega (Madison, WI, USA). The pGL4-hNRP1 promoter and pGL4-hNRP2 promoter plasmids were constructed by inserting the human NRP1 promoter region (−632 to −1 bp, relative to the transcription start site [TSS]) and human NRP2 promoter region (−481 to 25 bp, relative to the TSS) into the pGL4.16 vector, respectively. MH7A cells were transfected with plasmid DNA using ScreenFect A plus (FUJIFILM Wako Pure Chemical Co.) according to the manufacturer’s instructions. For knockdown experiments, drug selection using puromycin (InvivoGen, Hong Kong, China) was performed after transfection.

### 2.9. Synthesis of Recombinant S Protein

Recombinant S1 protein was constructed using a cell-free protein synthesis system with wheat germ ribosomal RNA. S1 protein (amino acid residues 318–685) was fused with glutathione S-transferase (GST) using the Robotic Protein Synthesizer Protemist DT, as previously described [26] (see Supplementary Figure S1). The C-terminal amino acid sequence “RRAR” was used, as both NRP1 and NRP2 bind to this motif [10]. All procedures were performed at CellFree Sciences (Yokohama, Japan).

### 2.10. S Protein Binding In Vitro Assay

MH7A cells or fibroblast-like synoviocytes (FLS) were seeded into a 96-well plate and cultured in Roswell Park Memorial Institute 1640 (RPMI 1640; FUJIFILM Wako Pure Chemical Co., Osaka, Japan) medium or Dulbecco’s modified Eagle’s medium (DMEM; FUJIFILM Wako Pure Chemical Co.) containing 10% fetal bovine serum (FBS; Sigma-Aldrich) and penicillin/streptomycin (FUJIFILM Wako Pure Chemical Co.) for 48 h. The cells were stimulated with TNFα (50 ng/mL), IL-1β (10 ng/mL), and recombinant S1 protein (500 ng/mL) for 24 h. A CoraLite 647-conjugated GST-tagged polyclonal antibody (Proteintech, Tokyo, Japan) was added to the cells, which were incubated for 1 h; fluorescence was then measured using SpectraMax iD3 (Molecular Devices, San Jose, CA, USA). Next, the CellTiter-Blue Cell Viability Assay Kit (Promega, Madison, WI, USA) was added to the cells, which were incubated for 1 h; fluorescence was then measured using SpectraMax iD3. The capacity of recombinant S1 protein to bind to MH7A cells or FLS was measured with compensation for cell viability.

### 2.11. SARS-CoV-2 Infection of MH7A Cells

The SARS-CoV-2/JP/Hiroshima-46059T/2020 strain (B.1.1, GISAID accession ID: EPI_ISL_6289932), which was isolated in Hiroshima, was used. For virus stock preparation, the infected cell culture medium was collected 48 h post-infection, clarified by low-speed centrifugation, and filtered through a 0.45 µm filter. Viral infectivity was measured using the standard TCID50 assay, as previously described [27]. MH7A cells treated with or without TNFα (50 ng/mL) and IL-1β (10 ng/mL) for 24 h were infected with SARS-CoV-2 at an input multiplicity of 10 and were maintained for 24 h. The culture medium was then collected, and the SARS-CoV-2 genome in the medium was quantified using the SARS-CoV-2 Direct Detection quantitative real-time PCR (RT–qPCR) Kit (Takara Bio, Kusatsu, Japan) and the Roche LightCycler II (Roche Diagnostics, Basel, Switzerland). All experiments involving SARS-CoV-2 were performed at the Biosafety Level 3 (BSL3) facility of Hiroshima University.

### 2.12. Statistical Analysis

All statistical analyses were conducted using Student’s *t*-test and Dunnett’s test. The results were analyzed and processed using the GraphPad Prism9 software (GraphPad, Inc., La Jolla, CA, USA).

## 3. Results

### 3.1. SARS-CoV-2 Host Receptor Gene Expression in Inflamed Synovial Tissues

**Figure 1:**
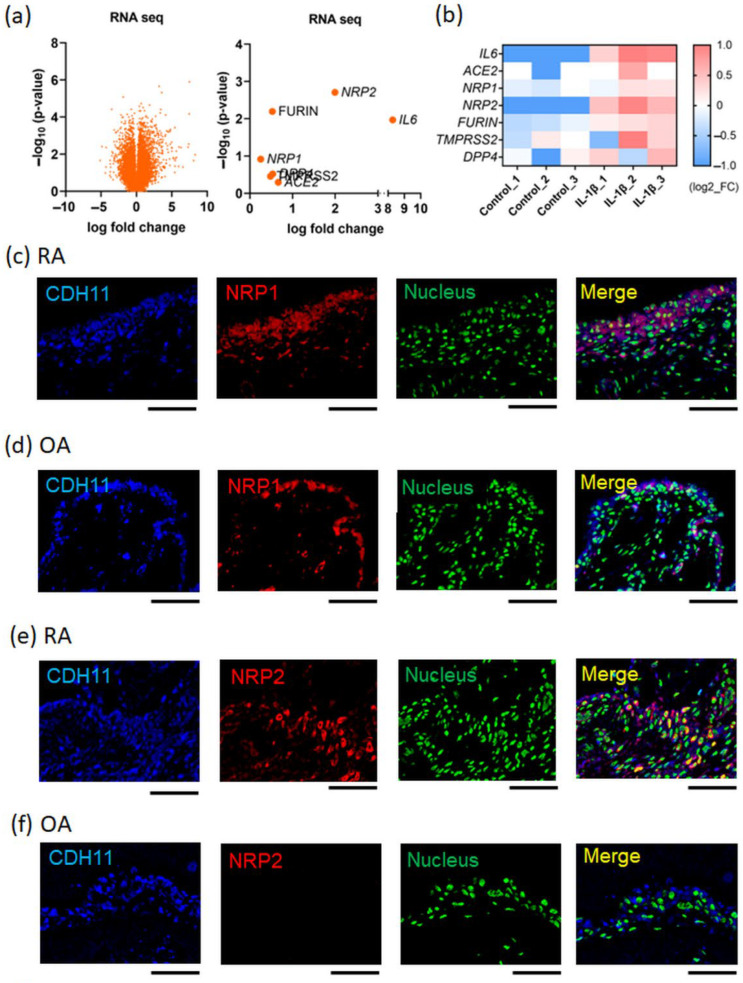

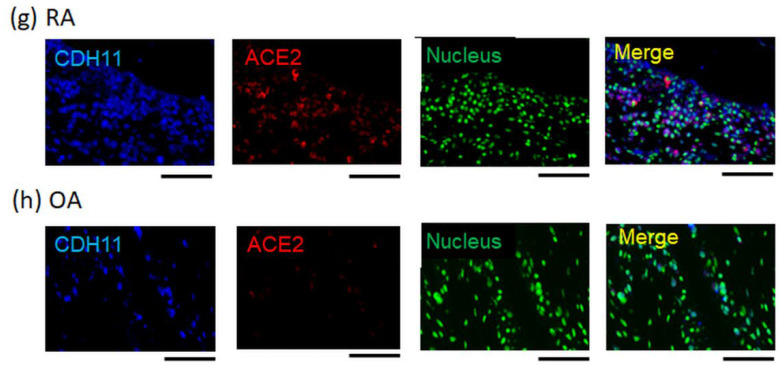
SARS-CoV-2 host receptor gene expression in inflamed synovial tissues. (**a**,**b**) Next-generation sequencing (RNA-seq) analysis for IL-1β (10 ng/mL)-stimulated fibroblast-like synoviocytes (FLS), which were extracted from rheumatoid arthritis (RA) patients’ synovial tissues (*n* = 3). (**a**) Volcano plot (**left** figure panel). The **right** figure displays the parameters of SARS-CoV-2 infection-related genes. (**b**) Heat map. SARS-CoV-2 infection-related genes were extracted from the RNA-seq dataset. (**c**–**h**) Dual-fluorescent immunohistochemistry (IHC) images conducted for formalin-fixed paraffin-embedded (FFPE) synovial tissues collected from RA and OA patients. The secondary antibodies were used: Alexa Fluor 405-conjugated anti-goat immunoglobulin G (IgG) and Alexa Fluor 647-conjugated anti-rabbit IgG. Dual-fluorescent IHC were merged with anti-CDH11 (blue), which was a surface marker of FLS, nuclear staining (green; acridine orange), and certain anti-host receptors (red). (**c**,**e**,**g**) RA patients’ synovial tissues. (**d**,**f**,**h**) Osteoarthritis (OA) patients’ synovial tissues. (**c**,**d**) Anti-NRP1 antibody (red). (**e**,**f**) Anti-NRP2 antibody (red). (**g**,**h**) Anti-ACE2 antibody (red). Representative images are shown. Scale bar = 100 µm.

Activation of the NLRP3 inflammasome, which results in the production of active IL-1β, occurs during the early stages of RNA virus infection; it can also be induced by peripheral blood mononuclear cells from patients with moderate or severe COVID-19 [22]. First, to investigate the association between IL-1β and the expression of SARS-CoV-2 host receptor genes, primary cultured FLS were stimulated with IL-1β and then analyzed using RNA-seq. The results showed that the expression of some host receptor genes, such as *NRP2*, was upregulated by IL-1β stimulation (Figure 1a,b). The normalized counts of *NRP2* (the mean ± the standard error of the mean) in the control and IL-1β-stimulated groups were 1939 ± 60 and 7793 ± 810, respectively. To analyze the expression patterns of host receptors under inflammatory conditions in vivo, immunohistochemistry (IHC) was performed on synovial tissue specimens. Samples from RA patients’ synovial tissues, which contain activated FLS (CDH11^+^) and numerous inflammatory cells, were used for inflammatory testing, while those from the OA patients’ synovial tissues, which contain inactivated FLS (CDH11^+^), were used as the noninflammatory control. IHC analysis revealed that NRP1, NRP2, and ACE2 were expressed in CDH11^+^ FLS in the synovial tissue (Figure 1c–h). The expression of NRP2 and ACE2 was upregulated in the RA synovial tissue compared with that in the OA synovial tissue (Figure 1e–h). In contrast, NRP1 expression was slightly higher in the RA synovial tissue than in the OA synovial tissue (Figure 1d,e). TMPRSS2 expression showed little difference between RA and OA synovial tissues (Supplementary Figure S2). Thus, host receptors for SARS-CoV-2 are expressed in FLS, and some of their expression is upregulated under inflammatory conditions, including under IL-1β stimulation.

### 3.2. Identification of Cytokines That Can Enhance NRP1 and NRP2 Expressions

**Figure 2:**
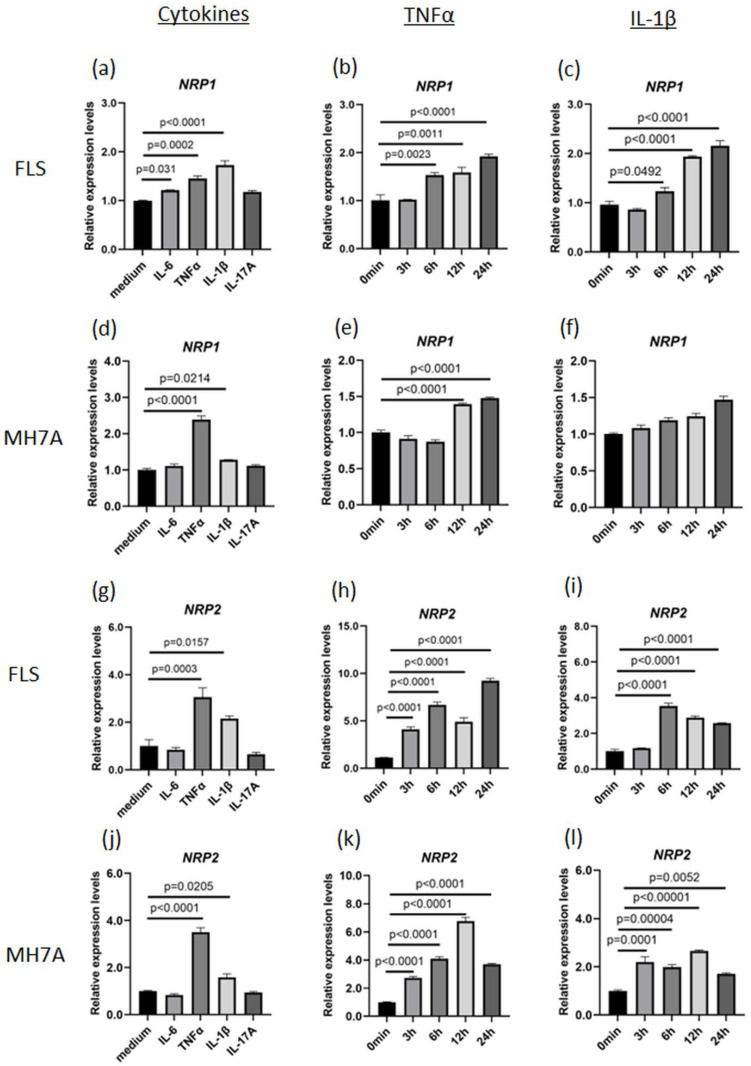
TNFα upregulates *NRP1* and *NRP2* expression. (**a**–**c**) *NRP1* expression in fibroblast-like synoviocytes (FLS) collected from patients with rheumatoid arthritis (RA). (**a**) Stimulation with TNFα (50 ng/mL), IL-1β (10 ng/mL), IL-6 (50 ng/mL with 50 ng/mL sIL-6R), and IL-17A (50 ng/mL) for 24 h. (**b**) Time-dependent stimulation with TNFα. (**c**) Time-dependent stimulation with IL-1β. (**d**–**f**) *NRP1* expression in MH7A cells. (**d**) Stimulation with TNFα, IL-1β, IL-6, and IL-17A. (**e**) Time-dependent stimulation with TNFα for 24 h. (**f**) Time-dependent stimulation with IL-1β. (**g**–**i**) *NRP2* expression in FLS was measured using RT–qPCR. (**g**) Stimulation with TNFα, IL-1β, IL-6, and IL-17A for 24 h. (**h**) Time-dependent stimulation with TNFα. (**i**) Time-dependent stimulation with IL-1β. (**j**–**l**) *NRP2* expression in MH7A cells. (**j**) Stimulation with TNFα, IL-1β, IL-6, and IL-17A for 24 h. (**k**) Time-dependent stimulation with TNFα. (**l**) Time-dependent stimulation with IL-1β. All expression levels were measured using RT–qPCR, and statistical analysis was performed using Dunnett’s test. Data represent the mean ± the standard error of the mean (*n* = 3). Data shown are representative results from several independent experiments.

To investigate whether NRP1 or NRP2 expression was upregulated by humoral factors, primary cultured FLS and MH7A cells (an immortalized FLS cell line) were stimulated with various proinflammatory cytokines. *NRP1* expression increased in FLS stimulated by TNFα or IL-1β (Figure 2a–c) and in MH7A cells stimulated by TNFα (Figure 2d–f). Similarly, NRP2 expression in FLS and MH7A cells increased following TNFα or IL-1β stimulation (Figure 2g–l). The alteration magnitude of *NRP2* expression levels induced by TNFα (about 5 to 10 times from baseline) were higher than those of NRP1 (about 1.5 times from baseline). RNA-seq data of FLS stimulated with IL-1β showed that *NRP1* expression was higher than *NRP2* expression under the cytokine-free condition, while IL-1β-induced *NRP2* expression reached a level comparable to *NRP1* expression (Supplementary Figure S3). This data revealed that NRP1 was constitutively expressed in both inflammatory and non-inflammatory conditions, while NRP2 was inducibly upregulated in the presence of proinflammatory cytokines. In addition, the existence of ACE2 is essential for SARS-CoV-2 infection. IL-1β stimulation also increased *ACE2* expression, similar to the findings in a study using IL-6 [23] (Supplementary Figure S4a). Therefore, several proinflammatory cytokines, including TNFα and IL-1β, could simultaneously upregulate or maintain the expression levels of *NRP1*, *NRP2*, and *ACE2* in human fibroblasts.

### 3.3. NF-κB p65 Transcription Factor Can Induce NRP2 Expression

**Figure 3:**
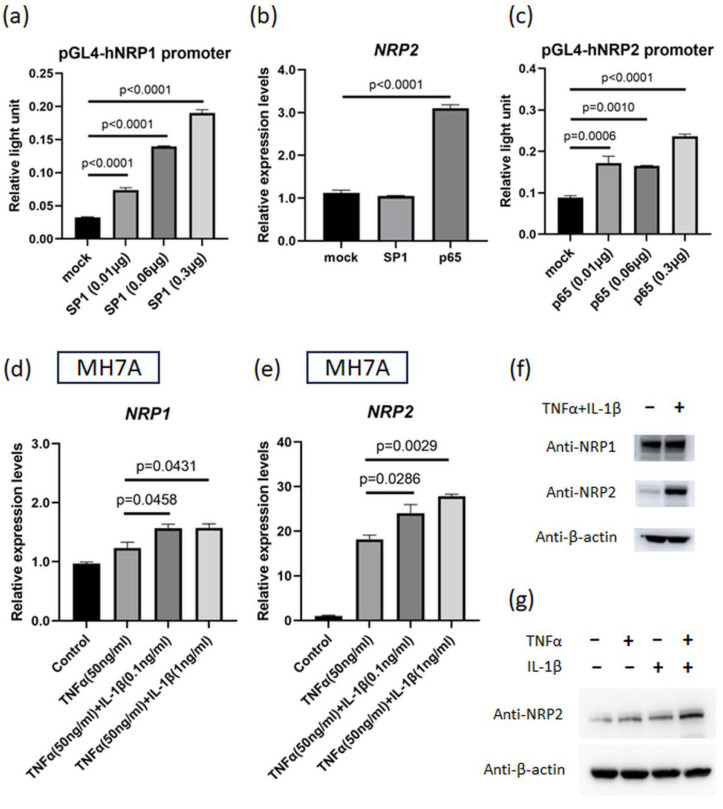

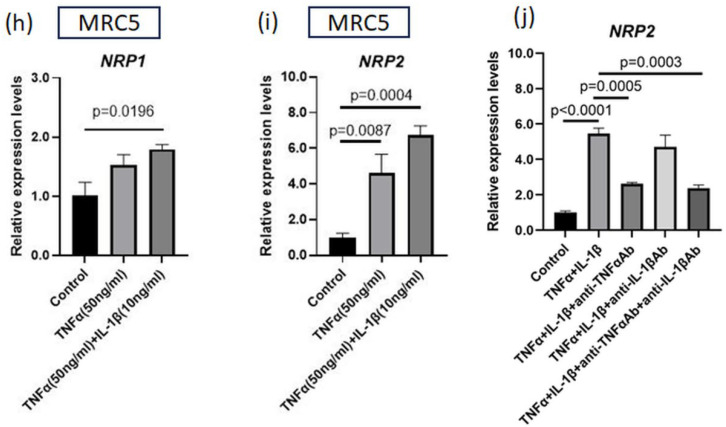
IL-1β plus TNFα treatment upregulates *NRP1* and *NRP2* expressions that are induced by transcription factors. (**a**) Luciferase assay for *NRP1* promoter region. Reporter plasmids and SP1 plasmid (or control vector) were simultaneously transfected into MH7A cells (*n* = 3). (**b**) *NRP2* expression in MH7A cells transfected with SP1 or NF-κB (p65) plasmids (*n* = 3). (**c**) Luciferase assay for *NRP2* promoter region. Reporter plasmids and p65 plasmid (or control vector) were transfected into MH7A cells (*n* = 3). (**d**,**e**) *NRP1* and *NRP2* expressions were measured using RT–qPCR. Cultured MH7A cells were stimulated with both TNFα (50 ng/mL) and IL-1β (various concentrations) (*n* = 3). (**d**) *NRP1* expression. (**e**) *NRP2* expression. (**f**,**g**) NRP1 and NRP2 levels were detected using Western blotting. Harvested MH7A cells were stimulated with or without TNFα (50 ng/mL) and IL-1β (10 ng/mL). (**h**,**i**) *NRP1* and *NRP2* expressions were measured using RT–qPCR. Cultured immortalized MRC5 cells were stimulated with both TNFα (50 ng/mL) and IL-1β (10 ng/mL) (*n* = 4). (**j**) Cultured MH7A cells were stimulated with TNFα (50 ng/mL) and IL-1β (1 ng/mL) and inhibited with anti-TNFα antibody (5 µg/mL) and anti-IL-1β antibody (1 µg/mL) (*n* = 3). (**a**–**e**,**h**–**j**) Data represent the mean ± the standard error of the mean and were calculated using Dunnett’s test.

The increases in NRP1 and NRP2 expression induced by proinflammatory cytokines suggest the presence of upstream transcription factors. Regarding NRP1 expression, SP1 is known to be a major transcription factor [28]. The results of luciferase assay on the promoter region of *NRP1* (−632 to −1 bp, relative to the TSS) revealed that SP1 enhanced *NRP1* promoter activity, which is consistent with previous reports (Figure 3a and Supplementary Figure S5). Moreover, according to the JASPAR database (http://jaspar2014.genereg.net; accessed on 26 January 2023), the promoter region of *NRP2* contains several NF-κB-binding motifs. NF-κB is among the downstream transcription factors of TNFα- and IL-1β-induced signaling. In our study, *NRP2* expression was enhanced by the overexpression of NF-κB (p65) but not by that of SP1 (Figure 3b), and luciferase assays using the promoter region of *NRP2* (−481 to 25 bp, relative to the TSS) demonstrated that its activity was enhanced by NF-κB (p65) (Figure 3c). Additionally, we investigated the major transcription factors of ACE2 and found that *ACE2* expression was increased by the overexpressed SP1 (Supplementary Figure S4b). These results revealed that SP1 and NF-κB are critical in controlling the expression of host receptor genes.

### 3.4. NRP2 Expression Is Elevated by TNFα and IL-1β Co-Stimulation

We next investigated the effect of TNFα and IL-1β co-stimulation on NRP1 and NRP2 expression in MH7A cells. The expression of both *NRP1* and *NRP2* under this co-stimulation was higher than that under the stimulation of TNFα alone (Figure 3d,e). Western blotting results showed that the amplitude of the change in NRP2 expression was much higher than that of NRP1 expression, and TNFα and IL-1β synergistically upregulated NRP2 expression (Figure 3f,g). We also evaluated the effects of these cytokines on a lung fibroblast cell line (immortalized MRC5 cells). TNFα and IL-1β co-stimulation increased the NRP2 expression levels on these cells (Figure 3h,i). Subsequently, we examined which cytokines were more important for *NRP2* expression using blocking antibodies against cytokines. As a consequence, the addition of anti-TNFα antibody had a greater inhibitory effect on *NRP2* expression than anti-IL-1β antibody (Figure 3j), indicating that TNFα dominantly regulated NRP2 expression in the inflammatory condition.

### 3.5. SARS-CoV-2 Infection and Proliferation under Inflammatory Conditions

**Figure 4:**
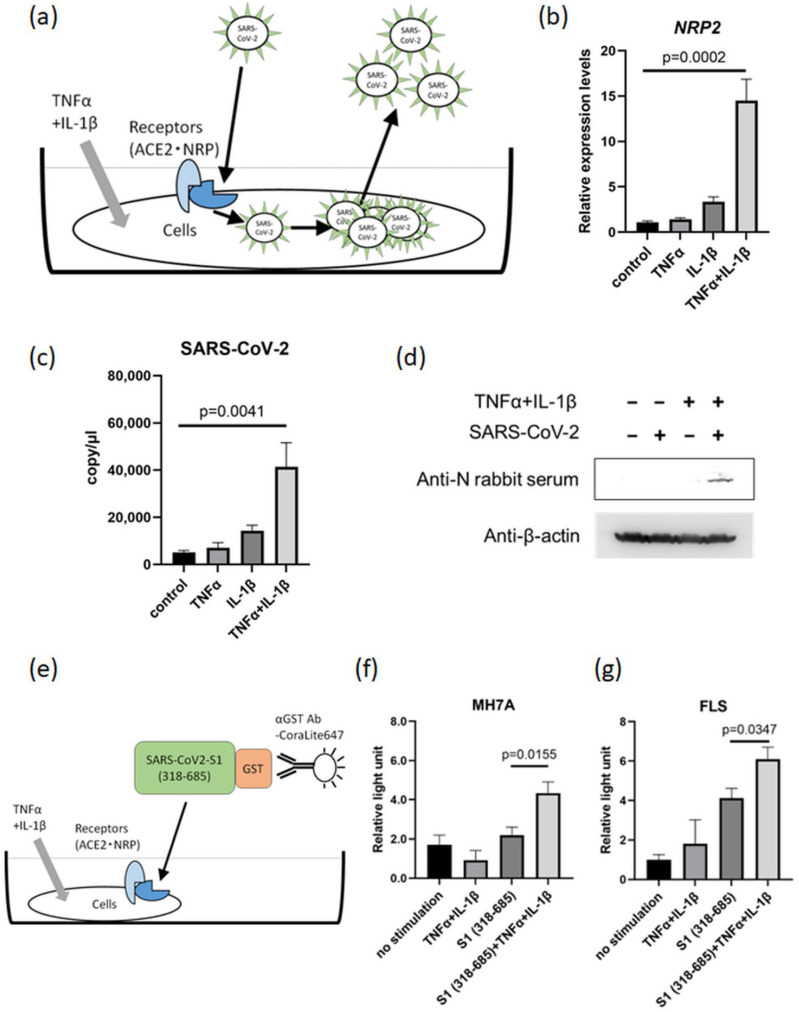
TNFα and IL-1β co-stimulation promotes SARS-CoV-2 proliferation. (**a**) Scheme of the roles of ACE2 and NRP in SARS-CoV-2 proliferation following TNFα and IL-1β co-stimulation. (**b**) *NRP2* expression in MH7A cells stimulated with TNFα (50 ng/mL) + IL-1β (10 ng/mL) under SARS-CoV-2-infected conditions (*n* = 3). (**c**) The copy number of SARS-CoV-2 in the supernatant from MH7A cells stimulated with TNFα and IL-1β (*n* = 3). (**d**) The intracellular Nucleocapsid protein in SARS-CoV-2-infected MH7A cells detected via Western blotting. Harvested MH7A cells were stimulated with or without TNFα (50 ng/mL) and IL-1β (10 ng/mL). (**e**) Scheme of the detection method for the binding between SARS-CoV-2 spike (S) protein and cells (MH7A and FLS) under TNFα and IL-1β co-stimulation. (**f**,**g**) SARS-CoV-2 S protein was added to MH7A cells stimulated with TNFα (50 ng/mL) and IL-1β (10 ng/mL), and fluorescence intensity analysis was conducted (*n* = 5). (**f**) MH7A cells. (**g**) Primary cultured FLS. (**b**,**c**) Measurement using RT–qPCR. Data represent the mean ± the standard error of the mean and were calculated using Dunnett’s test.

To investigate the effect of the inflammatory environment on SARS-CoV-2 infection, MH7A cells were stimulated with both TNFα and IL-1β and infected with SARS-CoV-2 (Figure 4a). As a result, MH7A co-stimulation with TNFα and IL-1β markedly increased the number of SARS-CoV-2 copies in the supernatant, in correlation with the increased expression of *NRP2* (Figure 4b,c). Moreover, the intracellular nucleocapsid protein of SARS-CoV-2 was also detected in MH7A cells co-stimulated with TNFα and IL-1β (Figure 4d). These data indicated that inflammatory cytokines are capable of promoting SARS-CoV-2 replication. Next, we evaluated the capacity of the SARS-CoV-2 S1 protein to bind to the host cell surface in an inflammatory environment using a recombinant S1 protein (Figure 4e). The results showed that the presence of TNFα and IL-1β increased the binding of the S1 protein to both MH7A cells and FLS (Figure 4f,g). Therefore, TNFα and IL-1β stimulation can increase both the proliferation of SARS-CoV-2 and the binding of the SARS-CoV-2 S protein to the host cell surface.

### 3.6. Proinflammatory Cytokine-Induced NRP2 Expression Contributes to Enhancement of Viral Proliferation

**Figure 5:**
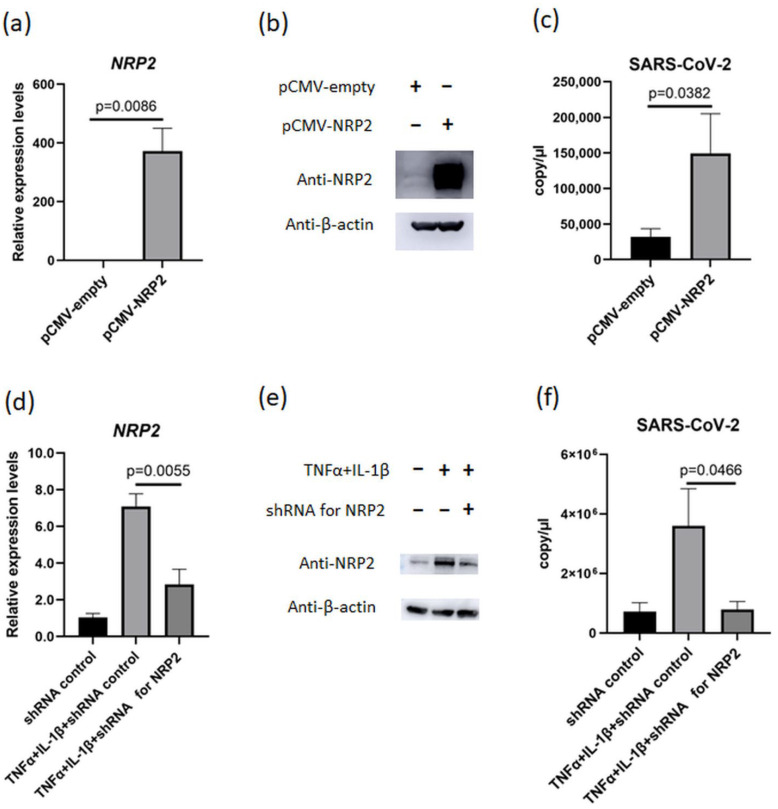
NRP2 contributes to SARS-CoV-2 proliferation in the presence of TNFα and IL-1β. (**a**,**b**) NRP2 expression measured using (**a**) RT–qPCR (*n* = 3) and (**b**) Western blotting after transfection of pCMV-empty or pCMV-NRP2 plasmids into MH7A cells. (**c**) The copy number of SARS-CoV-2 in the supernatant of MH7A cells transfected with or without NRP2 overexpression (*n* = 3). (**d**) *NRP2* expression was measured via RT–qPCR after transfection of anti-NRP2 shRNA and stimulation with TNFα (50 ng/mL) and IL-1β (10 ng/mL) (*n* = 3). (**e**) Western blotting after transfection of shRNA plasmids against NRP2 into MH7A cells and stimulation with TNFα and IL-1β. (**f**) The copy number of SARS-CoV-2 in the supernatant from MH7A cells transfected with shRNA plasmids against NRP2 and stimulated with TNFα and IL-1β (*n* = 3). Data represent the mean ± the standard error of the mean and were calculated using Dunnett’s test.

To certify the contribution of NRP2 in SARS-CoV-2 proliferation, we investigated the relationship between NRP2 expression levels and viral proliferation. First, NRP2 expression plasmid was introduced in MH7A cells, and these cells were infected with SARS-CoV-2 (Figure 5a,b). The culture supernatant of NRP2-overexpressing MH7A had a considerably increased copy number of SARS-CoV-2 (Figure 5c). Next, we performed knockdown experiments. *NRP2* expression increased in MH7A cells in the presence of TNFα and IL-1β, whereas this increase was suppressed by anti-NRP2 short hairpin (sh)RNA (Figure 5d,e). In the inflammatory environment, SARS-CoV-2 proliferation was markedly decreased in parallel with *NRP2* expression suppression (Figure 5f). These data indicated that NRP2 contributes to SARS-CoV-2 proliferation under inflammatory conditions.

## 4. Discussion

SARS-CoV-2 infects host cells via the binding of its surface-expressed S protein to the host receptor ACE2, which is highly expressed in the epithelial cells of the small intestine and oral cavity and is slightly expressed in vascular nasal mucosal epithelial cells and type II alveolar epithelial cells [29,30]. Moreover, NRP1 and NRP2 are expressed in many endothelial cells in the nasal cavity and lungs as well as in neurons [31]. S protein activation requires Furin-mediated cleavage, which exposes the C-terminal RRAR sequence that has a high affinity for NRP1 and NRP2 [10]. NRP1 reportedly assists the binding of the S protein to ACE2 and promotes SARS-CoV-2 entry into host cells. Our results showed that NRP2 expression level is substantially lower in non-inflammatory conditions. Under physiological conditions, the ability of NRP2 to assist in binding the S protein to ACE2 is highly limited. In contrast, in the inflammatory conditions, NRP2 expression level is equal to or greater than NRP1. Not only NRP1, but also NRP2 are supposed to assist binding of S protein to ACE2 under pathological conditions. Furin is abundantly expressed in oral epithelial cells and fibroblasts [32]. This Furin-mediated modification of cell-attached SARS-CoV-2 could facilitate viral entry via NRPs. In neural tissues, SARS-CoV-2 infiltration has been detected, possibly due to NRP1 expression in the vascular endothelium and neurons, although the expression of ACE2 and TMPRSS2 is considered low [33]. The role of NRP2 in COVID-19 pathophysiology has seldom been reported, though NRP2 can reportedly bind to the S protein [10]. In this study, we demonstrated that the inflammatory environment contributes to SARS-CoV-2 proliferation through not only ACE2 and NRP1 but also NRP2.

SARS-CoV-2 is recognized by pattern-recognition receptors on innate immune cells, eventually producing proinflammatory cytokines, such as IL-6, TNFα, and IL-1β. These cytokines have been detected in patients with severe COVID-19 [11,34]. S protein-bound ACE2 activates a disintegrin and metalloprotease 17 (ADAM17), which enhances the inflammatory response via activation of the TNFα and IL-6 receptors and contributes to the induction of a cytokine storm [35,36]. These findings imply that proinflammatory cytokines directly promote COVID-19 pathogenesis. The results of our study indicate that these cytokines enhance NRP2 expression and affect SARS-CoV-2 proliferation. In particular, the anti-TNFα antibody dominantly suppresses NRP2 expression *in vitro*. These results were consistent with a report from the Global Rheumatology Alliance stating that TNFα inhibitors-treated patients with rheumatic diseases had a reduced risk of hospitalization from COVID-19 [21]. Therefore, anti-cytokine therapy, especially anti-TNFα therapy, may inhibit the viral proliferation of SARS-CoV-2 and reduce infection-mediated organ damage. Furthermore, NRP2 has potential as a target for novel therapeutics against COVID-19. Since NRP2 is a human-derived protein that cannot be mutated, anti-NRP2 therapy, such as monoclonal antibodies, may provide stable effectiveness. Moreover, anti-NRP2 antibody might not produce side effects under noninflammatory conditions, in which the expression of NRP2 is low.

In our presented data, NRP1 was upregulated by SP1, which is compatible with previous reports. SP1 contributes to NRP1 expression in human glioma cells [28]. However, the major transcription factors involved in NRP2 and ACE2 expression have never been reported. Our study demonstrates that NRP2 expression is regulated by NF-κB, whereas ACE2 expression is regulated by SP1. NF-κB is regulated in the signaling pathway via receptors of proinflammatory cytokines, such as TNFα and IL-1β. SP1 expression is reportedly upregulated by these cytokines in nucleus pulposus cells [37]. These findings indicate that the expression of major host receptors, NRP1, NRP2, and ACE2, is regulated by transcription factors located downstream of proinflammatory cytokine-induced signaling pathways.

This study had some limitations. This study revealed that the inflammatory environment promotes SARS-CoV-2 proliferation in MH7A cells; however, this result has not been confirmed in other cells. SARS-CoV-2 invades the bloodstream through mucous membranes and can affect multiple organs. Accordingly, all organs of the body, including the synovial tissues, should be investigated. Further studies are warranted to determine which organ is influenced by the inflammatory environment in SARS-CoV-2 proliferation.

## 5. Conclusions

SARS-CoV-2 proliferation is promoted in an inflammatory environment and is correlated with inducible NRP2 expression. Thus, NRP2 affects SARS-CoV-2 proliferation in coordination with NRP1 and ACE2 under inflammatory conditions and may serve as a novel therapeutic target for COVID-19 treatment.

## Data Availability

The datasets used and/or analyzed during the current study are available from the corresponding author upon reasonable request. The RNA-seq raw data are available in the NCBI Sequence Read Archive with accession number PRJNA915654.

## Supplementary Materials

The following supporting information can be downloaded at: https://www.mdpi.com/article/10.3390/v15071498/s1, Supplementary Figure S1: Detection of recombinant S1 protein via Western blotting; Supplementary Figure S2: Dual-fluorescent IHC for RA and OA synovial tissues; Supplementary Figure S3: Comparison between NRP1 and NRP2 expression levels in IL-1β-stimulated FLS; Supplementary Figure S4: IL-1β and SP1 upregulate ACE2 expression; Supplementary Figure S5: Design of plasmids for luciferase assay.
