# Supplementary material for: Tumor Necrosis Factor and Interleukin-1β Upregulate NRP2 Expression and Promote SARS-CoV-2 Proliferation
Source: Viruses. 2023 Jul 3;15(7):1498. doi: 10.3390/v15071498 (PMC10383177; doi:10.3390/v15071498)
Supplement: Supplementary file 1 [file viruses-15-01498-s001.zip › viruses-2434701-supplementary.pdf]

Supplementary Figure S1

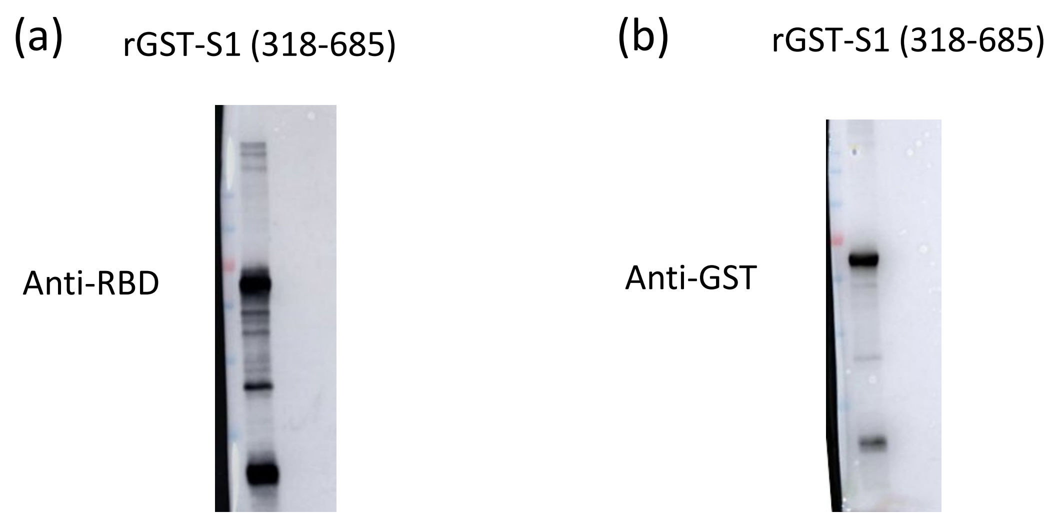

Supplementary Figure S1. Detection of recombinant S1 protein via western blotting

(a, b) S protein (S1; 318-685) was detected using (a) anti-RBD and (b) anti-GST antibodies in western blot analyses. RBD, receptor-binding domain; GST, glutathione-S-transferase.

Supplementary Figure S2

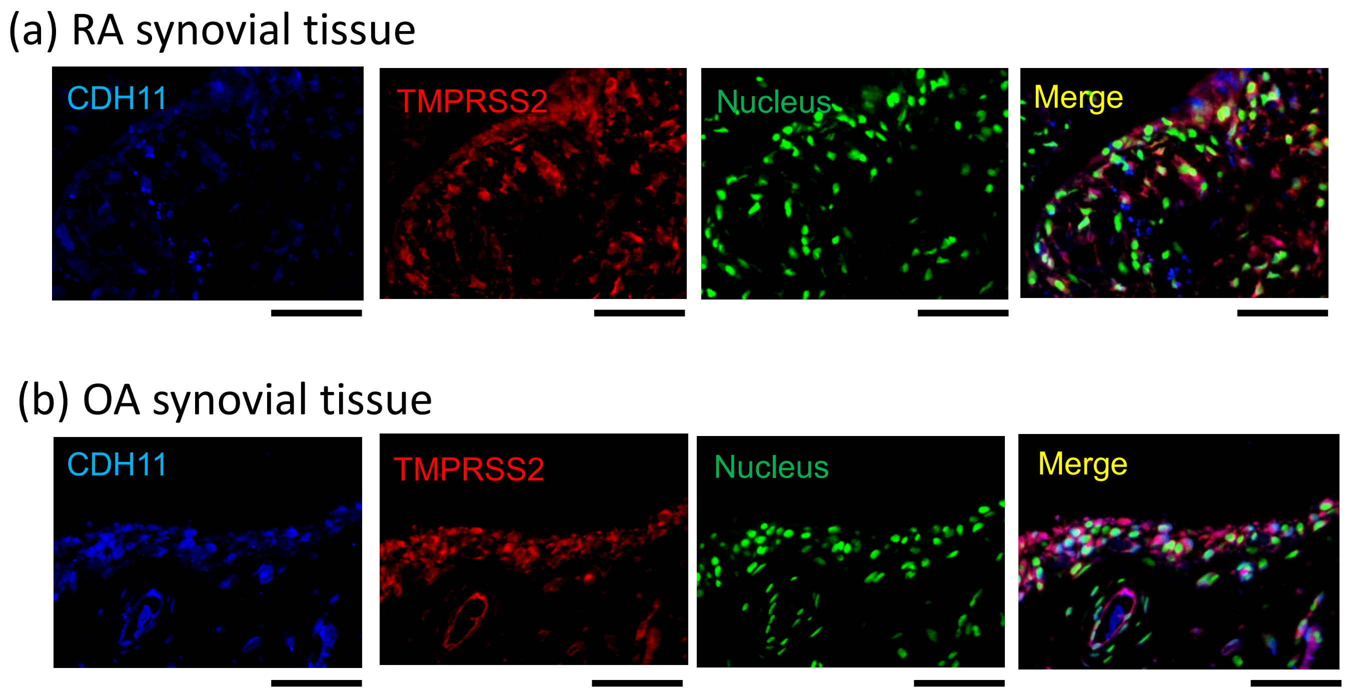

Supplementary Figure S2. Dual-fluorescent IHC for RA and OA synovial tissues

Dual-fluorescent IHC images of the synovia of (a) RA and (b) OA. Anti-CDH11 (blue), nuclear staining (green), and anti-TMPRSS2 (red) antibodies. Black scale bar = 100 µm.

Supplementary Figure S3

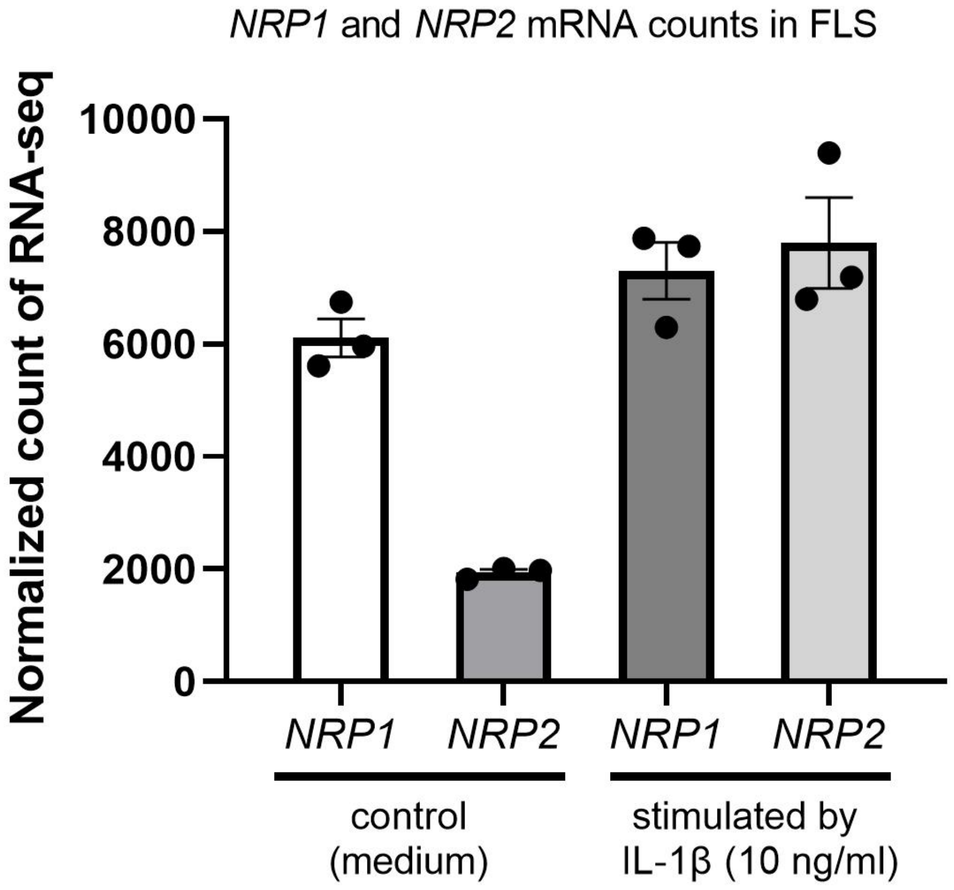

Supplementary Figure S3. Comparison between NRP1 and NRP2 expression levels in IL-1β-stimulated FLS

Fibroblast-like synoviocytes (FLS) were stimulated with IL-1β and subsequently analyzed using RNA-seq.

The normalized read count data revealed that *NRP1* exhibited constitutive expression, irrespective of the presence or absence of IL-1β stimulation. On the other hand, *NRP2* was identified as an inducible gene, being upregulated specifically in response to IL-1β stimulation.

Furthermore, under inflammatory conditions, the expression levels of *NRP2* were found to be similar to those of *NRP1*.

Supplementary Figure S4

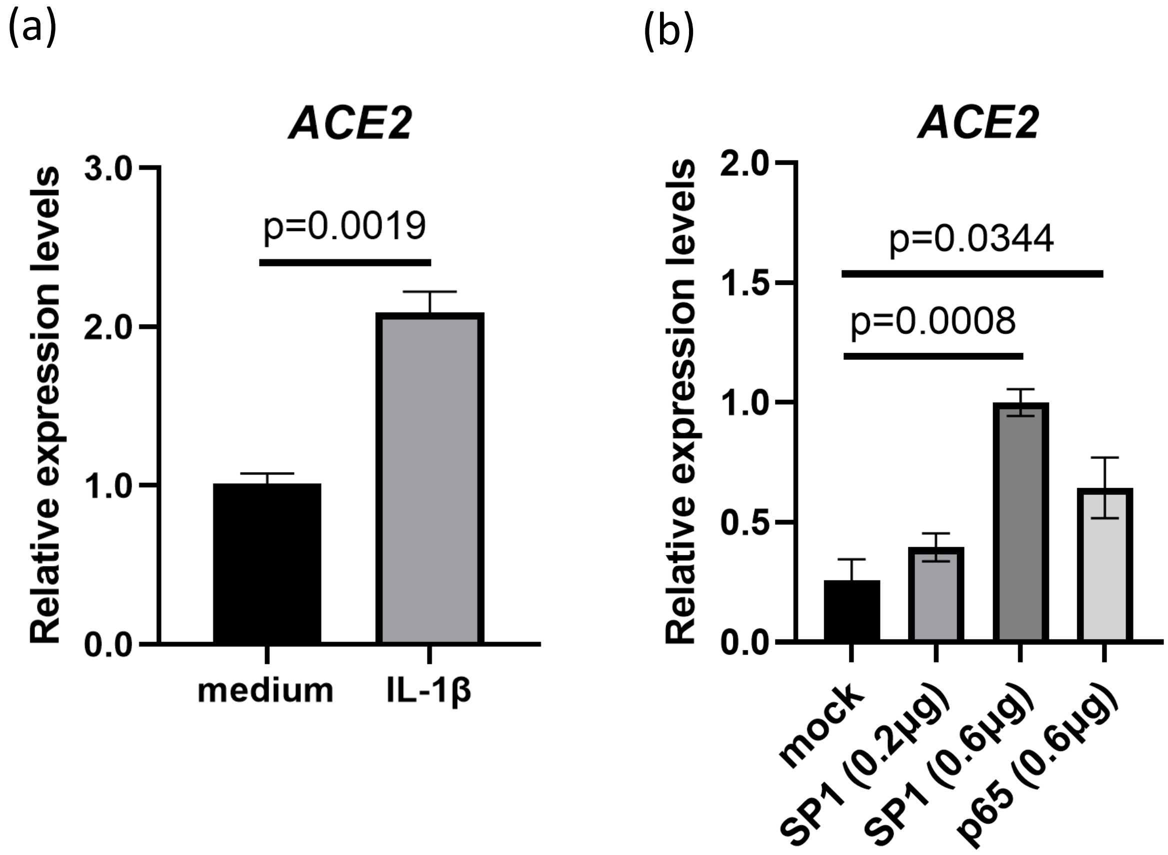

Supplementary Figure S4. IL-1β and SP1 upregulate ACE2 expression

(a) *ACE2* expression in MH7A cells stimulated with IL-1β (10 ng/mL). (b) *ACE2* expression in MH7A cells transfected with the SP1 and NF-κB (p65) plasmids. Data represent the mean ± the standard error of the mean and were calculated using Student's t-test or Dunnett's test. SP1, specificity protein 1.

Supplementary Figure S5

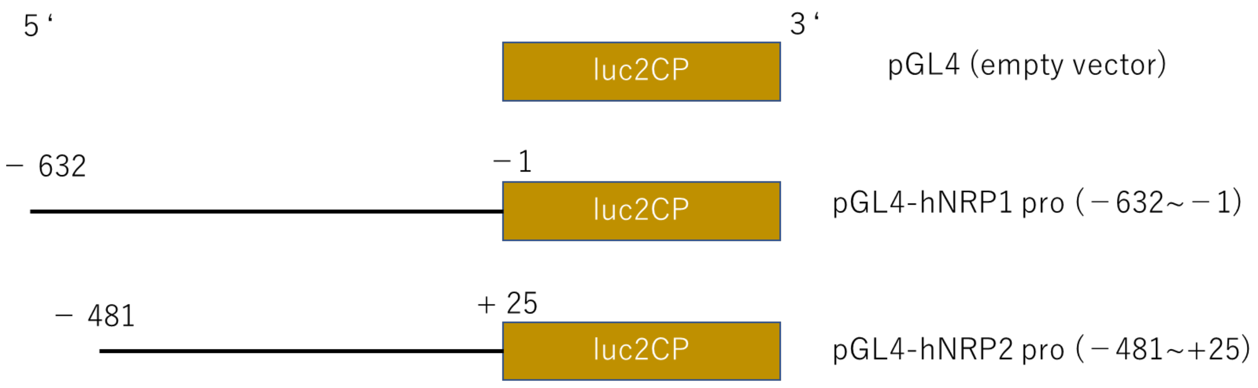

Supplementary Figure S5. Design of plasmids for luciferase assay

Scheme of the pGL4-basic plasmid (upper) and the pGL4 plasmids (pGL4-hNRP1 promoter and pGL4-hNRP2 promoter) containing luciferase (luc2CP)-linked NRP1 and NRP2 promoter sequences (middle and bottom, respectively).
